# Liver Dysfunction Assessed by Model for End-Stage Liver Disease Excluding INR (MELD-XI) Scoring System Predicts Adverse Prognosis in Heart Failure

**DOI:** 10.1371/journal.pone.0100618

**Published:** 2014-06-23

**Authors:** Satoshi Abe, Akiomi Yoshihisa, Mai Takiguchi, Takeshi Shimizu, Yuichi Nakamura, Hiroyuki Yamauchi, Shoji Iwaya, Takashi Owada, Makiko Miyata, Takamasa Sato, Satoshi Suzuki, Masayoshi Oikawa, Atsushi Kobayashi, Takayoshi Yamaki, Koichi Sugimoto, Hiroyuki Kunii, Kazuhiko Nakazato, Hitoshi Suzuki, Shu-ichi Saitoh, Yasuchika Takeishi

**Affiliations:** 1 Department of Cardiology and Hematology, Fukushima Medical University, Fukushima, Japan; 2 Department of Advanced Cardiac Therapeutics, Fukushima Medical University, Fukushima, Japan; Kurume University School of Medicine, Japan

## Abstract

**Aims:**

Liver dysfunction due to heart failure (HF) is often referred to as cardiac or congestive hepatopathy. The composite Model for End-Stage Liver Disease excluding INR (MELD-XI) is a robust scoring system of liver function, and a high score is associated with poor prognosis in advanced HF patients with a heart transplantation and/or ventricular assist device. However, the impact of MELD-XI on the prognosis of HF patients in general remains unclear.

**Methods and Results:**

We retrospectively analyzed 562 patients who were admitted to our hospital for the treatment of decompensated HF. A MELD-XI score was graded, and patients were divided into two groups based on the median value of MELD-XI score: Group L (MELD-XI <10, *n* = 289) and Group H (MELD-XI ≥10, *n* = 273). We compared all-cause mortality and echocardiographic findings between the two groups. In the follow-up period (mean 471 days), 104 deaths (62 cardiac deaths and 42 non-cardiac deaths) were observed. The event (cardiac death, non-cardiac death, all-cause death)-free rate was significantly higher in group L than in group H (logrank *P*<0.05, respectively). In the Cox proportional hazard analysis, a high MELD-XI score was found to be an independent predictor of cardiac deaths and all-cause mortality in HF patients. Regarding echocardiographic parameters, right atrial and ventricular areas, inferior vena cava diameter, and systolic pulmonary artery pressure were higher in group H than in group L (*P*<0.05, respectively).

**Conclusions:**

The MELD-XI scoring system, a marker of liver function, can identify high-risk patients with right heart volume overload, higher pulmonary arterial pressure and multiple organ failure associated with HF.

## Introduction

Liver dysfunction often exists in heart failure (HF) as cardiac or congestive hepatopathy, and is associated with adverse clinical outcomes in advanced HF patients. [1]–[5] Cardio-hepatic syndrome, a condition characterized by the development of congestive hepatopathy and subsequent cirrhosis in patients with advanced HF, has been recognized. [6] Recently, the composite Model for End-Stage Liver Disease (MELD) scoring series, with types such as MELD (including total bilirubin, creatinine, prothrombin time-international ratio (INR)), MELDNa (MELD with sodium), and MELD-XI (MELD excluding INR) have been developed. They are established scoring systems of liver or hepato-renal function, and a high score is associated with poor prognosis, not only in patients having undergone liver transplantation [7] but also advanced HF considered heart transplantation and/or ventricular assist device. [1], [8]–[10] In HF patients, MELD scoring series indicates multiple organ dysfunction secondary to impaired cardiac function. It has been reported that MELD scoring series were strong predictors for mortality than each values alone (total bilirubin, creatinine, INR, sodium).[1] Since HF patients often receive anticoagulant therapy, liver function evaluation by MELD-XI seems to have results superior to an MELD score. [10] A high MELD-XI score is associated with a poor prognosis in HF patients who have had a heart transplantation, and/or have a ventricular assist device. [1], [8]–[10] However, the impact of MELD-XI on the prognosis of general HF patients remains unclear. On another note, right ventricular systolic function, [11], [12] and pulmonary arterial pressure, [12], [13] are the reported predictors of prognosis in HF patients.

Therefore, the aim of the present study was to investigate the association of liver function (MELD-XI score) with 1) prognosis (including cardiac, non-cardiac, and all-cause mortality) in general HF patients, and 2) cardiac function (especially right heart function).

## Methods

### Subjects and study protocol

We retrospectively searched for 621 consecutive patients who were hospitalized at Fukushima Medical University hospital for the treatment of decompensated HF between 2009 and 2012. The diagnosis of decompensated HF was defined based on the Framingham criteria. [14] Patients with acute coronary syndrome, viral hepatitis, hepatic tumors, bile duct disease, end-stage kidney disease and/or dialysis, pulmonary thromboembolism, and primary pulmonary hypertension were excluded. Finally, we analyzed 562 patients. Liver function was estimated by the MELD-XI formula. As previously reported, MELD-XI was calculated as: (5.11×log (total bilirubin)+11.76×log (creatinine)+9.44); if variables with a value of <1 were given the value of 1. [10] These patients were divided into two groups based on the median value of MELD-XI score in our study subjects: Group L (MELD-XI <10, *n* =  289) and Group H (MELD-XI ≥10, *n* =  273). We compared the clinical features and results from several examinations of both groups, such as general laboratory tests and echocardiography, performed upon hospital admission. Hypertension was defined as the recent use of antihypertensive drugs, or a systolic blood pressure ≥140 mmHg, and/or a diastolic pressure ≥90 mmHg. Diabetes was defined as the recent use of insulin or antidiabetic drugs, a fasting blood glucose value of ≥126 mg/dL, and/or a hemoglobinA_1_c value of ≥6.5%. Dyslipidemia was defined as the recent use of cholesterol-lowering drugs, a triglyceride value of ≥150 mg/dL, a low-density lipoprotein cholesterol value of ≥140 mg/dL, and/or a high-density lipoprotein cholesterol value of <40 mg/dL. Estimated glomerular filtration rate (eGFR) was measured by the Modification of Diet in Renal Disease formula. [15] Anemia was defined as hemoglobin of <12.0 g/dl in females and <13.0 g/dl in males. [16] Pulmonary hypertension was defined as an estimated systolic pulmonary artery pressure (SPAP) ≥36 mmHg at rest in the absence of pulmonary outflow obstruction by echocardiography. [17] Patients were followed up for cardiac death, non-cardiac death, and all-cause mortality. Non-cardiac death included death due to stroke, respiratory failure, infection, sepsis, cancer, digestive haemorrhage, and etc. Status and dates of deaths were obtained from the patients' medical records. If these data were unavailable, status was ascertained by a telephone call to the patient's referring hospital physician. Written informed consent was obtained from all study subjects. The study protocol was approved by the ethical committee of Fukushima Medical University.

### Echocardiography

Echocardiography was performed blindly by an experienced echocardiographer using the standard techniques. Echocardiographic parameters investigated included left ventricular (LV) volume, left ventricular ejection fraction (LVEF), left atrial volume, the ratio of early transmitral flow velocity to mitral annular velocity (mitral valve E/e'), inferior vena cava diameter, SPAP, right atrial end systolic area, right ventricular (RV) area, right ventricular fractional area change (RV-FAC), tissue Doppler-derived tricuspid lateral annular systolic velocity (tricuspid valve S'), and the ratio of the peak transtricuspid velocity during early diastole to the peak tricuspid valve annular velocity during early diastole (tricuspid valve E/e'), etc.[17] The LVEF was calculated using a modification of the Simpson's method. Mitral valve E/e' was calculated by transmitral Doppler flow and tissue Doppler imaging. SPAP was calculated by adding the right atrial pressure (estimated by the diameter and collapsibility of the inferior vena cava) to the systolic trans tricuspid pressure gradient. [12], [17] The RV-FAC, defined as (end diastolic area-end systolic area)/end diastolic area x 100, is a measure of right ventricular systolic function. [17] Tricuspid valve E/e' was calculated by transtricuspid Doppler flow and tissue Doppler imaging. All recordings were performed on ultrasound systems (ACUSON Sequoia, Siemens Medical Solutions USA, Inc., Mountain View, CA, USA).

### Statistical analysis

Normally distributed data are presented as mean ± SD, non-normally distributed data are presented as median (interquartile range), and categorical variables are expressed as numbers and percentages. Characteristics between the two groups were compared using the independent Student's *t*-test for normally distributed data and the Mann-Whitney U- test for non-normally distributed data, whereas the chi-square test was used for categorical variables. Kaplan-Meier method was used for presenting the event-free rate and the logrank test was used for initial comparisons. Univariate and multivariate Cox proportional hazard analyses were used to analyze predictors of events and adjusting for confounding factors. To prepare for potential confounding, we introduced the following factors, known to affect the risk of worsening heart failure, cardiac death or all-cause mortality in HF patients and parameters of liver function: age, gender, systolic blood pressure, presence of ischemic etiology, atrial fibrillation, anemia, reduced LVEF (<50%), and higher (more than the median value of) BNP, aspartate aminotransferase, alkaline phosphatase, gamma-glutamyl transferase, sodium, C-reactive protein, and MELD-XI score. Univariate parameters with a *P*-value of <0.10 were included in the multivariate analysis. A value of *P* < 0.05 was considered significant for all comparisons. These analyses were performed using a statistical software package (SPSS ver. 21.0, IBM, Armonk, NY).

## Results

As shown in *Tables 1* and *2*, comparisons of clinical features revealed that Group H had: 1) lower total protein, albumin, and cholinesterase levels, 2) higher alkaline phosphatase and gamma-glutamyl transferase levels, 3) larger right atrial and ventricular areas, inferior vena cava diameter, SPAP, and tricuspid valve E/e', and 4) lower left ventricular ejection fraction. In contrast, RV systolic function (RV-FAC, tricuspid valve S') did not differ between the two groups. In summary, Group H had poorer nutrition, a higher cholestatic state, right heart volume overload, higher pulmonary arterial pressure, and lower LV systolic function.

**Table 1:** Comparisons of Group L and Group H clinical features.

	Group L (*n* = 289)	Group H (*n* = 273)	*P*-value
Age (years)	67.2±14.7	68.1±13.7	0.437
Male gender (*n*, %)	148 (51.2)	191 (70.0)	<0.001
Body mass index (kg/m^2^)	23.1±4.2	23.3±4.1	0.496
Systolic blood pressure (mmHg)	121.3±17.9	117.8±19.7	0.125
Diastolic blood pressure (mmHg)	72.1±11.2	72.4±12.0	0.868
Heart rate (bpm)	67.5±13.9	71.6±14.8	0.018
Ischemic etiology (*n*, %)	76 (26.3)	67 (24.5)	0.698
Co-morbidity			
Hypertension (*n*, %)	203 (70.2)	205 (75.1)	0.219
Diabetes (*n*, %)	91 (31.5)	107 (39.2)	0.064
Dyslipidemia (*n*, %)	208 (72.0)	200 (73.3)	0.777
Atrial fibrillation (*n*, %)	92 (31.8)	118 (43.2)	0.007
Anemia (*n*, %)	120 (41.5)	168 (61.5)	<0.001
Medications			
ACE inhibitors/ARB (*n*, %)	217 (75.1)	191 (70.0)	0.186
β-blockers (*n*, %)	209 (72.3)	218 (79.9)	0.068
Diuretics (*n*, %)	170 (58.8)	165 (60.4)	0.731
Aldosterone blockers (*n*, %)	117 (40.5)	114 (41.8)	0.797
Warfarin/anti coagulation (*n*, %)	144 (49.8)	146 (53.5)	0.399
Laboratory data			
White blood cell (×10^3^/µl)	7.30±3.38	7.52±3.35	0.454
Platelet (×10^3^/µl)	18.1±6.5	19.6±7.5	0.019
Hemoglobin (g/dl)	12.7±2.1	11.9±2.7	<0.001
Prothrombin time-international ratio	1.39±0.64	1.50±0.86	0.135
BNP (pg/ml) †	236.9 (457)	517.1 (1069)	<0.001
Blood urea nitrogen (mg/dl)	24.4±13.3	23.0±12.9	0.239
Creatinine (mg/dl)	0.80±0.18	2.05±1.05	<0.001
eGFR (ml/min/1.73 cm^2^)	71.0±18.1	40.4±21.9	<0.001
Uric acid (mg/dl)	6.8±2.1	6.5±2.3	0.146
Total protein (g/dl)	7.0±0.8	6.8±0.8	0.014
Albumin (g/dl)	3.7±0.6	3.4±0.6	<0.001
Total bilirubin (mg/dl)	0.8±0.3	1.2±0.7	<0.001
Direct bilirubin (mg/dl)	0.1±0.0	0.2±0.1	<0.001
Aspartate aminotransferase (U/L)	51.6±17.5	52.5±18.7	0.703
Alanine aminotransferase (U/L)	36.5±26.3	43.4±18.6	0.345
Alkaline phosphatase (U/L)	258.1±121.3	297.3±150.8	<0.001
Gamma-glutamyl transferase (U/L)	59.4±32.9	76.8±41.9	0.033
Cholinesterase (U/L)	273.3±82.6	227.0±78.9	<0.001
Sodium (mEq/l)	138.8±4.5	138.9±3.8	0.787
C-reactive protein (mg/dl) †	0.35 (1)	0.37 (1)	0.661

ACE, angiotensin-converting enzyme; ARB, angiotensin II receptor blocker; BNP, B-type natriuretic peptide; eGFR, estimated glomerular filtration.

† Data are presented as median (interquartile range).

**Table 2:** Comparisons of echocardiographic data.

	Group L (*n* = 289)	Group H (*n* = 273)	*P*-value
Left ventricular end-diastolic volume (ml)	109.4±59.2	125.2±59.3	0.005
Left ventricular end-systolic volume (ml)	58.2±43.8	72.5±44.4	0.001
LVEF (%)	50.6±15.1	44.7±15.2	<0.001
Left atrial volume (ml)	76.6±31.6	98.8±43.3	0.001
Mitral valve inflow E/A	1.02±0.58	1.27±0.79	0.002
Mitral valve inflow early wave deceleration time (msec)	212.8±95.1	208.1±111.0	0.642
Mitral valve e' (cm/sec)	6.4±2.8	6.3±5.3	0.701
Mitral valve E/e'	14.0±7.8	17.9±9.9	<0.001
Inferior vena cava diameter (mm)	14.7±4.9	16.4±5.3	0.001
SPAP (mmHg)	27.7±12.9	31.1±13.2	0.013
Pulmonary hypertension (*n*, %)	84 (44.7)	99 (58.2)	0.011
Right atrial end systolic area (cm^2^)	18.6±9.8	22.7±17.4	0.027
Right ventricular area-diastolic (cm^2^)	16.3±6.1	18.4±8.1	0.030
Right ventricular area-systolic (cm^2^)	9.5±4.1	11.3±5.7	0.009
RV-FAC (%)	42.2±11.9	42.9±16.8	0.749
Tricuspid valve S' (cm/sec)	10.3±4.8	9.1±3.9	0.146
Tricuspid valve inflow E/A	1.13±0.49	1.04±0.35	0.322
Tricuspid valve E/e'	5.3±2.3	7.5±5.5	0.009

LVEF, left ventricular ejection fraction; Mitral valve inflow E/A, ratio of early to late transmitral peak flow velocities; Mitral valve E/e', ratio of the peak transmitral velocity during early diastole to the peak mitral valve annular velocity during early diastole; SPAP, systolic pulmonary artery pressure; RV-FAC, right ventricular fractional area change; Tricuspid valve S', tissue Doppler-derived tricuspid lateral annular systolic velocity; Tricuspid valve inflow E/A, ratio of early to late transtricuspid peak flow velocities, Tricuspid valve E/e', ratio of the peak transtricuspid velocity during early diastole to the peak tricuspid valve annular velocity during early diastole.

**Figure 1:**
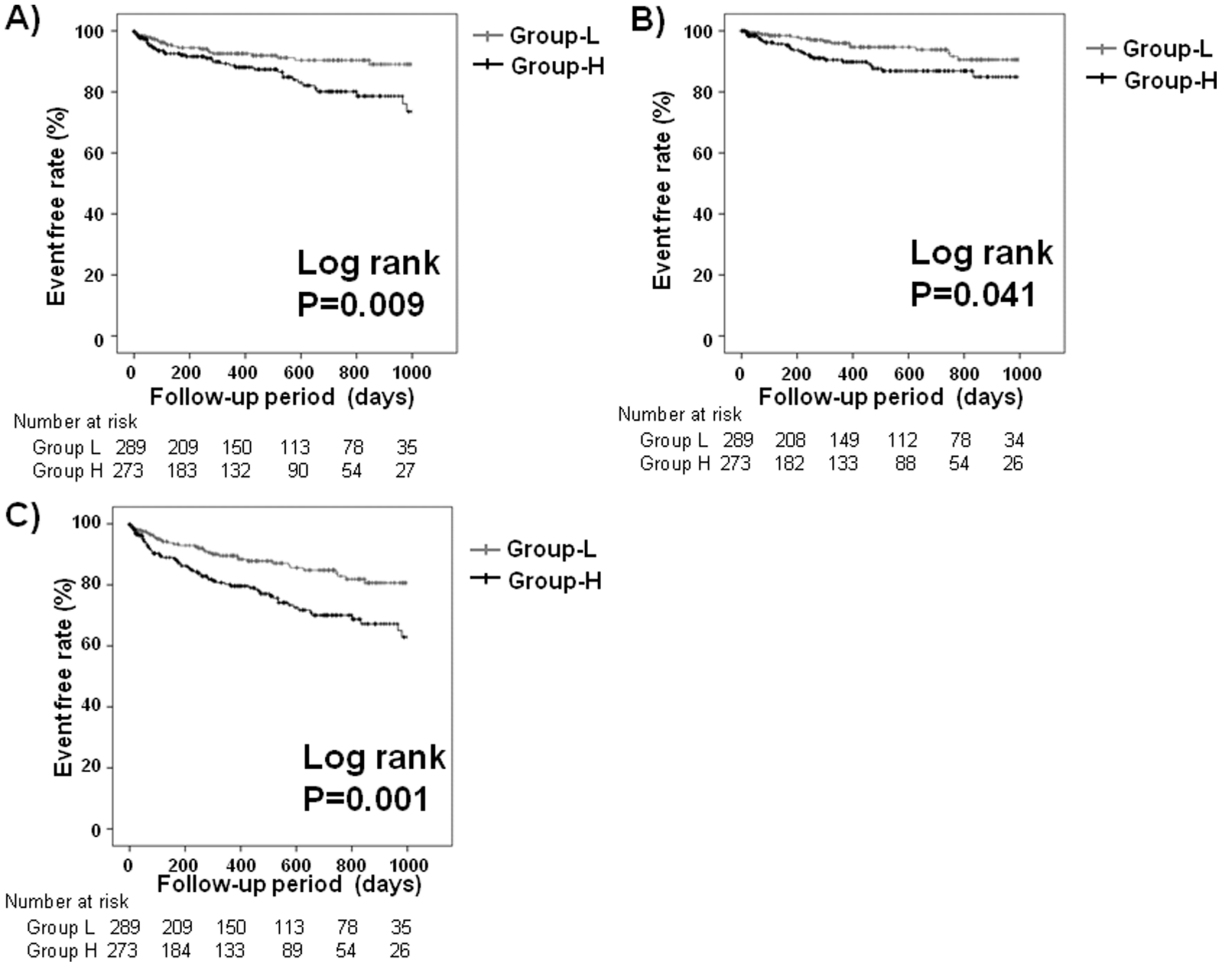
Kaplan-Meier analysis for A) cardiac death, B) non-cardiac death, and C) all-cause mortality between group L and group H.

During the follow-up period (mean 471 days), there were 62 cardiac deaths and 42 non-cardiac deaths. Details of cardiac and non-cardiac deaths were as follows: heart failure deaths (*n* = 50), ventricular fibrillation (*n* = 12), cancer (*n* = 12), respiratory failure and/or pneumonia (*n* = 11), infection/sepsis (*n* = 6), stroke (*n* = 5), digestive haemorrhage (*n* = 3), renal failure (*n* = 2), and others (*n* = 3). As shown in *Figure 1*, the event (cardiac death, non-cardiac death, all-cause mortality)-free rate was significantly lower in Group H than in Group L (*P*<0.05, respectively).

To examine prognostic factors in HF patients, the Cox proportional hazard model was used (*Tables 3* *,* *4* *,* *5*). With respect to cardiac death in HF patients (*Table 3*), reduced LVEF (HR 2.234, 95% CI 1.142–4.371, *P* = 0.019), aspartate aminotransferase (HR 1.856, 95% CI 1.021–3.375, *P* = 0.043), and MELD-XI (HR 2.052, 95% CI 1.085–3.879, *P* = 0.027) were independent predictors. With respect to non-cardiac death in HF patients (*Table 4*), MELD-XI was a predictor in univariate analysis, however, MELD-XI was not an independent predictor in mutivariate analysis. Age was an independent predictor for non-cardiac death (HR 1.065, 95% CI 1.030–1.101, *P*<0.001). With respect to all-cause mortality in HF patients (*Table 5*), age (HR 1.029, 95% CI 1.008–1.049, *P* = 0.005), reduced LVEF (HR 1.625, 95% CI 1.009–2.617, *P* = 0.046) and MELD-XI (HR 1.650, 95% CI 1.025–2.654, *P* = 0.036) were independent predictors. In summary, a high MELD-XI score was an independent predictor of cardiac death and all cause mortality.

**Table 3:** Cox proportional hazard model of cardiac death in HF.

Risk factor		Univariate			Multivariate	
	HR	95% Cl	*P*-value	HR	95% Cl	*P*-value
Age	1.008	0.901–1.028	0.401			
Male gender	1.150	0.687–1.926	0.595			
Systolic blood pressure	0.977	0.954–1.122	0.118			
Ischemic etiology	1.167	0.661–2.059	0.595			
Atrial fibrillation	0.892	0.526–1.513	0.671			
Anemia	1.487	0.879–2.515	0.139			
Reduced LVEF (LVEF<50%)	2.370	1.369–4.103	0.002	2.234	1.142–4.371	0.019
BNP	2.148	1.145–4.028	0.017	1.465	0.764–2.810	0.250
Aspartate aminotransferase	2.326	1.375–3.937	0.002	1.856	1.021–3.375	0.043
Alkaline phosphatase	1.333	0.798–2.226	0.272			
Gamma-glutamyl transferase	1.004	0.580–1.739	0.998			
Sodium	0.958	0.564–1.629	0.875			
C-reactive protein	1.093	0.663–1.803	0.728			
MELD-XI	1.973	1.178–3.305	0.010	2.052	1.085–3.879	0.027

BNP, B-type natriuretic peptide; LVEF, left ventricular ejection fraction; MELD-XI, model for end-stage liver disease excluding prothrombin time-international ratio.

**Table 4:** Cox proportional hazard model of non-cardiac death in HF.

Risk factor		Univariate			Multivariate	
	HR	95% Cl	*P*-value	HR	95% Cl	*P*-value
Age	1.071	1.038–1.105	<0.001	1.065	1.030–1.101	<0.001
Male gender	0.560	0.305–1.029	0.062	0.499	0.248–1.003	0.057
Systolic blood pressure	0.989	0.959–1.020	0.491			
Ischemic etiology	0.936	0.437–2.003	0.864			
Atrial fibrillation	1.398	0.755–2.591	0.287			
Anemia	1.768	0.886–3.528	0.106			
Reduced LVEF (LVEF<50%)	1.014	0.537–1.916	0.966			
BNP	2.065	1.011–4.221	0.047	1.762	0.855–3.634	0.125
Aspartate aminotransferase	0.874	0.470–1.626	0.670			
Alkaline phosphatase	1.594	0.840–3.027	0.154			
Gamma-glutamyl transferase	0.852	0.425–1.710	0.652			
Sodium	1.262	0.663–2.402	0.478			
C-reactive protein	0.991	0.541–1.817	0.977			
MELD-XI	1.893	1.015–3.530	0.045	1.667	0.828–3.355	0.152

Abbreviations as in Table 3.

**Table 5:** Cox proportional hazard model of all-cause mortality in HF.

Risk factor		Univariate			Multivariate	
	HR	95% Cl	*P*-value	HR	95% Cl	*P*-value
Age	1.028	1.011–1.044	<0.001	1.029	1.008–1.049	0.005
Male gender	0.897	0.606–1.327	0.585			
Systolic blood pressure	0.982	0.963–1.145	0.132			
Ischemic etiology	1.124	0.717–1.762	0.611			
Atrial fibrillation	1.034	0.693–1.541	0.871			
Anemia	1.967	1.307–2.961	0.001	1.566	0.943–2.600	0.083
Reduced LVEF (LVEF<50%)	1.511	1.015–2.249	0.042	1.625	1.009–2.617	0.046
BNP	2.152	1.331–3.478	0.002	1.516	0.910–2.525	0.110
Aspartate aminotransferase	1.464	0.991–2.162	0.056	1.286	0.820–2.016	0.274
Alkaline phosphatase	1.382	0.924–2.066	0.115			
Gamma-glutamyl transferase	0.872	0.564–1.347	0.537			
Sodium	1.036	0.686–1.565	0.867			
C-reactive protein	1.015	0.688–1.498	0.940			
MELD-XI	1.961	1.312–2.931	0.001	1.650	1.025–2.654	0.036

Abbreviations as in Table 3.

## Discussion

To the best of our knowledge, the present study is the first to show the utility of MELD-XI scores for predicting detailed cardiac and non-cardiac deaths in general HF patients with regard to right heart function. It was found that MELD-XI was an independent predictor of cardiac death and all-cause mortality in general HF patients, whose conditions were associated with right heart volume overload and higher pulmonary arterial pressure.

HF results in a various abnormal liver functions, such as the elevation of serum bilirubin, alkaline phosphatase, ganma-glutamyl transferase, and alanine aminotransferase. The mechanism possibly responsible for liver dysfunction in HF is considered to be caused by hemodynamic influences: decreased hepatic blood flow originating from low cardiac output and increased hepatic venous pressure with subsequent atrophy of liver cells and edema of the peripheral area, both leading to hepatocellular hypoxia. [18] Previous hemodynamic data suggest that elevated central venous pressure and right atrial pressure may contribute to cholestatic abnormalities and impairment of hepatocyte function in patients with HF. [19], [20] Several metabolic processes of bilirubin, including secretion of direct bilirubin into bile, [21] are attenuated by hepatocellular hypoxia. In addition, biliary obstruction caused by elevated hepatic venous pressure leads to an increase of serum total bilirubin. Several studies so far have shown that serum bilirubin correlates with various hemodynamic and cardiac parameters, such as right atrial pressure, [19], [22] severity of tricuspid regurgitation, [19], [22] pulmonary artery wedge pressure, [5], [19] cardiac output, [5], [19] and LVEF. [22] In our study, HF with a high MELD-XI score was accompanied by poorer nutrition, cholestatic state, cardiac volume overload, and higher pulmonary arterial pressure. In contrast, RV systolic function did not differ between the two groups. It has been reported that RV systolic dysfunction is a predictor of cardiac events in HF. [11], [12] In addition, elevated central venous pressure [23] and pulmonary atrial pressure [12], [13], [24] are shown to be associated with an adverse prognosis in HF. Reactive post capillary pulmonary hypertension is a prognostic factor regardless of HF etiology or left ventricular systolic function. [24] It seems that the prognostic importance of MELD-XI score in our study, at least in part, reflects the greater elevations in central venous pressure, cardiac volume overload, and pulmonary hypertension.

Shinagawa *et al*. reported that high total bilirubin is an independent predictor of cardiac events in HF patients. [5] It has been reported that MELD and MELD-XI were independent predictors of all-cause mortality in advanced HF patients (HF patients after having received a ventricular assist device). [8], [10] Recently, it has been reported that MELD models were independent predictors of all-cause mortality in advanced HF patients (HF patients either considered for or having undergone heart transplantation). [1], [9] Our study differs from previous studies [1], [8]–[10] in many ways. For instance, we presented detailed all-cause death in general HF patients who received no ventricular assist device and/or heart transplantation. Importantly, we also showed the association with right heart function.

### Study limitations

Several limitations remain in the present study. First, it was a retrospective analysis of a single institution. The number of subjects was relatively small. Hence, prospective studies with a larger population are needed. However, diagnosis of HF was accurately made by our experienced cardiologists using the Framingham criteria. Second, we evaluated RV function and SPAP using echocardiography unless we used right heart catheterization. However, this is not routinely performed.

## Conclusions

A high MELD-XI score was an independent predictor of not only cardiac death but also all-cause mortality in HF patients. HF patients with high MELD-XI scores had 1) poorer nutrition, 2) a greater cholestatic state, and 3) right heart volume overload and higher pulmonary arterial pressure. These mechanisms may in part affect the adverse prognosis of HF patients with high MELD-XI scores.
